# Towards spill-free in-bag morcellation: a health failure mode and effects analysis

**DOI:** 10.1007/s00464-018-6284-z

**Published:** 2018-07-09

**Authors:** Lukas van den Haak, Anne C. van der Eijk, Evelien M. Sandberg, Gerard Peter G. M. Frank, Karin Ansink, Rob C. M. Pelger, Cor D. de Kroon, Frank Willem Jansen

**Affiliations:** 1 Department of Gynecology, Leiden University Medical Center, PO Box 9600, 2300 RC Leiden, The Netherlands; 2 Central Sterile Supply Department, Leiden University Medica Centre, PO Box 9600, 2300 RC Leiden, The Netherlands; 3 Operating Room Center, Leiden University Medica Centre, PO Box 9600, 2300 RC Leiden, The Netherlands; 4 Department of Urology, Leiden University Medical Center, PO Box 9600, 2300 RC Leiden, The Netherlands; 5 Department of BioMechanical Engineering, Delft University of Technology, PO Box 5, 2600 AA Delft, The Netherlands

**Keywords:** Hysterectomy, Laparoscopy, Morcellation, Myomectomy, Sarcoma

## Abstract

**Background:**

To assess potential risks of new surgical procedures and devices before their introduction into daily practice, a prospective risk inventory (PRI) is a required step. This study assesses the applicability of the Health Failure Mode and Effects Analysis (HFMEA) as part of a PRI of new technology in minimally invasive gynecologic surgery.

**Methods:**

A reference case was defined of a patient with presumed benign leiomyoma undergoing a laparoscopic hysterectomy or myomectomy including in-bag power morcellation; however, pathology defined a stage I uterine leiomyosarcoma. Using in-bag morcellation as a template, a HFMEA was performed. All steps of the in-bag morcellation technique were identified. Next, the possible hazards of these steps were explored and possible measures to control these hazards were discussed.

**Results:**

Five main steps of the morcellation process were identified. For retrieval bags without openings to accommodate instruments inside the bag, 120 risks were identified. Of these risks, 67 should be eliminated. For containment bags with openings 131 risks were identified of which 68 should be eliminated. Of the 10 causes most at risk to cause spillage, two can be eliminated by using appropriate bag materials. Myomectomy appears to be more at risk for residual tissue spillage compared to total hysterectomy.

**Conclusion:**

The HFMEA has provided important new insights regarding potential weaknesses of the in-bag morcellation technique, particularly with respect to hazardous steps in the morcellation process as well as requirements that bags should meet. As such, this study has shown HFMEA to be a valuable method that identifies and quantifies potential hazards of new technology.

A prospective risk inventory (PRI) assesses potential hazards of new surgical procedures and devices (henceforth called technology) before their introduction in daily practice [1]. However, prospectively assessing potential hazards of new and therefore unknown technology is challenging and methods are needed to aid this assessment.

The Failure Mode and Effects Analysis (FMEA) is a step-by-step method, performed by a group of people involved in the process, aiming to identify failure modes and their effects before new technology is introduced in daily practice. Failure modes are manners in which a process may fail, and the effects analysis examines the consequences of these failures. It was developed in 1949 to prospectively evaluate problems that might occur from malfunctioning of new military systems [2]. Due to its prospective nature, the conclusions are based on inductive reasoning. Nowadays, an FMEA is an important tool in safety and reliability engineering of consumer products, such as the car industry. The FMEA was adapted to also suit healthcare requirements: the Health Failure Mode and Effects Analysis (HFMEA). However, experience with this method as part of a PRI is limited [3, 4].

The goal of our study was to assess the applicability of the HFMEA for new technology in gynecology. We used the efficacy of in-bag morcellation, with respect to the prevention of tissue spillage during morcellation, as a template for this method. Currently in minimally invasive gynecologic surgery (MIGS), in-bag morcellation is nearly regarded as the new gold standard for morcellation procedures to overcome the past safety issues regarding the spread of potential malignant tissue. The results of in vitro tests regarding the efficacy of containing tissue are favorable and the clinical feasibility has been demonstrated [5–8]. However, the oncological safety has yet to be proven. Furthermore, comparative trials are no longer ethically feasible, and prospective clinical data will only become available in several years. In theory, the HFMEA procedure overcomes these shortcomings of standard research methodology and provides necessary safety information of the in-bag morcellation technique in its early phase of implementation in daily clinical practice.

## Materials and methods

The HFMEA was performed according to the instructions of the prospective risk analysis system developed by the Department of Veterans Affairs, National Center for Patient Safety [3].

The analysis consisted of 5 steps: choosing a subject, assembling a team for the analysis, describing the complete process of the subject of interest, performing a hazard analysis of the entire process, and finally resolving all serious hazards.

### Step 1: choosing the subject

To maintain a workable analysis of the subject of interest, it should be defined as precise as possible. This allows the number of actions and potential hazards to be limited to the ones that are most essential to the subject. For our study, a fictional case was defined of a patient with undiagnosed stage I uterine leiomyosarcoma, undergoing a laparoscopic hysterectomy (LH) or myomectomy (LM) including in-bag power morcellation for presumed benign leiomyoma. This scenario was chosen as in theory these patients are most likely harmed by the occurrence of spillage of malignant cells due to an immediate upstaging of the disease, resulting in a significantly worse prognosis. Our focus being on spillage, risks considered inherent to all (laparoscopic) surgical procedures were excluded from the scope of the present study.

### Step 2: assembling the team

A team is assembled representing most of the health care workers who are involved with the subject of interesting. An expert in the subject and a team leader are appointed. The expert helps explaining the process, and the team leader chairs the meetings. Novices to the process are also part of the team to promote an unbiased critical view of current practice.

### Step 3: describing the in-bag morcellation process

Next, the studied subject is graphically described in a flow diagram. The main steps are defined and then for each main step the sub-steps are identified. Two types of morcellation bags were assessed: regular specimen retrieval bags without openings to accommodate instruments thus consequently in need of iatrogenic breach of bag integrity, and containment bags equipped with openings to accommodate a camera or instrument. Furthermore, morcellation after LH and after LM was evaluated.

### Step 4: hazard analysis

For the complete process as determined in step 3, all possible hazards (the so-called failure modes, meaning how and why a process might fail) are identified. To rank these hazards from most to least serious, these failure modes are scored for their impact on patient health or safety (‘severity’) and their chance of occurring (‘probability’). By multiplying the scores for severity and probability, a hazard score is calculated: high scores correspond with serious hazards. Severity was classified according to the following 4 categories: recovery without intervention (1 point), intervention needed for recovery (2 points), permanent damage or loss of function (3 points), and death (4 points) [9] (Table 1). To maximize results of our analysis, any kind or amount of tissue spread or leakage was considered potentially lethal. ‘Probability’ was defined as occurring in less than 1000 procedures (1 point), between 1 in 100 and 1000 procedures (2 points), between 1 in 10 and 100 procedures (3 points), and 1 in 10 or more (4 points). A mean hazard score was calculated based on the individual scores provided by all participants. All participants calculated the hazard scores individually. Next, the scores were compared during the meetings and, in case of disagreement, discussed and adjusted to reach full agreement upon the final hazard score.

**Table 1:**
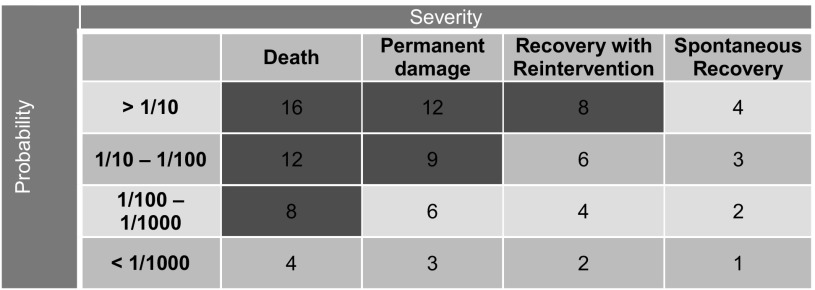
Probability and severity score

Red*—*hazard score ≥ 8 represents a safety issue and should be addressed

### Step 5: resolving serious hazards

The HFMEA decision tree is used to identify the failure modes that may cause safety issues and should therefore be eliminated [3]. In short, this decision tree identifies all hazards with a hazard score ≥ 8 as a potential threat that needs to be addressed (Red in Table 1). Readily apparent hazards and hazards that are already known and controlled can be exempted. In addition, the decision tree identifies all critical steps in the process (the so-called single point of weaknesses, meaning that the technique would fail if this single step fails) even with a hazard score < 8.

After identifying these hazards, recommendations were formulated in our final meeting to control the most hazardous steps of the in-bag morcellation procedure.

## Results

**Diagram 1:**
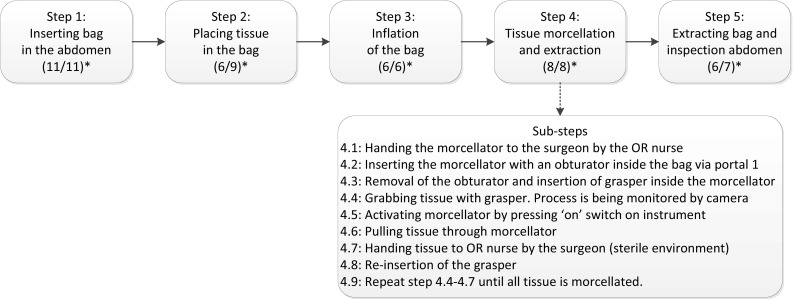
All main steps of the morcellation process. *Number of sub-steps for each main step: (retrieval bag without sleeves/containment bag with sleeves). Arrow *(*bottom*)*: sub-steps of main step 4 ‘tissue morcellation and extraction’

A total of 7 participants were selected for the HFMEA: 1 gynecologist-oncologist, 1 gynecologist specialized in minimally invasive gynecologic surgery (MIGS), 1 urologist specialized in minimally invasive surgery, 1 operating room (OR) nurse, 2 residents in gynecology, and 1 researcher in MIGS. The senior author acted as the expert on the in-bag morcellation process. An expert in surgical instrument safety with experience in HFMEA procedures (ACE) was appointed as team leader. To complete the five steps, six sessions of 2 h (in total 12 h) were needed, followed by additional discussion via email. During the first two sessions, five main steps of the morcellation process were identified. The first step is inserting the bag in the abdomen. This involves unwrapping the bag, handing it to the OR nurse and then to the surgeon, until the bag is correctly placed inside the abdomen. In step 2, the tissue is placed in the bag, meaning that the tissue is grabbed by surgical graspers and is manipulated inside the bag. In step 3, the bag is positioned for morcellation and inflated with CO2 gas. For specimen retrieval bags without extra openings, this also involves the iatrogenic puncture of the bag to accommodate surgical instrument inflation of the bag. In step 4, the tissue is morcellated and extracted until all tissue has been removed. Finally in step 5, the bag is retrieved from the abdomen and the abdomen is inspected for tissue residue. Diagram 1 demonstrates these steps, as well as the number of sub-steps that were found for each main step. As an example, the sub-steps of main step number 4 (morcellation and extraction of tissue) are described in full in the same diagram.

For regular retrieval bags without openings, 37 sub-steps and 120 failure modes were identified. Of these failure modes, 67 could be eliminated. For containment bags with openings, 41 sub-steps and 131 failure modes were identified. Of these, 68 were identified as possible hazards that could be eliminated.

In Table 2, for both bags the moments of the in-bag morcellation procedure most at risk for tissue spillage (meaning with the highest hazard scores of 16) are supplied. These moments consist mainly of steps where tissue fragments may spread throughout the abdomen by instruments that have come in contact with the morcellated tissue. Possible measures to control these risks are also proposed in Table 2. Hazards specific for myomectomy and additional hazards of retrieval bags without openings are highlighted in the same table. 4 out of 10 causes most at risk to cause spillage are specific for myomectomy and do not occur during hysterectomy. Two more causes can be eliminated by avoiding the iatrogenic puncture of retrieval bags without openings to accommodate the camera or morcellation instrument.

**Table 2:** Top hazard scores for both type of bags

Failure mode	Cause	Possible prevention
Contamination of the outside of bag	**Grasping with contaminated instruments**	**Inherent to myomectomy procedure**
	**Contact with exposed myoma**	
Contamination of surgical instruments before morcellation	**Grasping myoma**	
Contamination of the abdominal cavity, peritoneum, or abdominal wall	**Contact with the outside of the bag that was contaminated during main steps 1 and 2**	
	Spreading of remnant tissue by airflow of the pneumoperitoneum	Remove all tissue from abdomen/place all tissue in bag
Contamination of the camera and surgical instruments situated inside the bag	By tissue fragments being spread during morcellation	Clean camera and instruments before re-insertion into abdominal cavity after morcellation procedure
Contamination of surroundings and instrument table	By handling of the contaminated morcellator, contaminated camera, or instruments	Prevent contact as much as possible, use a different instrument table, and consider changing gloves in case of full contact
Occurrence of tissue spillage	Bag or tissue damage due to sharp graspers and other sharp instruments	Use appropriate non-sharp instruments
*Contamination of trocars*	*By contact with tissue inside bag when trocar is used to perforate bag*	*Avoid using regular bags without a pre-fabricated insert for camera*
*Contamination of the abdominal cavity, peritoneum, or abdominallwall*	*By leakage of tissue through perforation during extraction of the bag from the abdomen*	

Bold—hazards specific for myomectomy. Italic*—*additional specific hazards for regular bags in need of perforation before morcellation

## Discussion

Using in-bag morcellation as a template, our study assessed the applicability of the HFMEA method as part of a PRI for new technology. Although the in-bag morcellation technique is already widely used in daily clinical practice, the HFMEA revealed several possible weaknesses that should be addressed to further enhance the safety of this procedure.

All instruments (camera, graspers, portals) used inside the bag are a possible source for tissue spillage. In theory, when these instruments are later re-introduced into the abdominal cavity, they may unintentionally insert tissue remnants that were initially contained in the bag. Also, handling these instruments and placing them on the instrument table may contaminate the surgical team itself and other instruments. However, in case of sarcoma the oncological effect of minimal amounts of spread or leakage, henceforth called micro-spillage, into the abdominal cavity is unclear. For instance, in endometrial carcinoma the spread of malignant cells in the abdominal cavity via the fallopian tubes during hysteroscopy does not appear to negatively influence clinical outcome [10–12]. The same might apply to our findings. Nonetheless, in theory it seems appropriate to thoroughly clean or even change all instruments and surgical gloves after morcellation. In the same light, it became clear that myomectomy is at higher risk regarding tissue spread compared to hysterectomy. Tissue spillage during myomectomy has been demonstrated even without morcellation [13, 14]. Therefore in myomectomy, tissue may have spread both during and after the excision of the myoma, even before morcellation is performed. This does not occur after (total) hysterectomy, assuming that the integrity of the leiomyosarcomas is preserved. It follows that 4 out of 10 causes most at risk for spillage can be eliminated when comparing hysterectomy to myomectomy (Table 2). It should be stressed, however, that the meaning of micro-spillage is unclear and this study does not propose to abandon the minimally invasive removal of fibroids. Yet in this light, the possible consequences of micro-spillage should be further studied to reach a complete understanding of the risks of morcellation.

The second main hazard for contamination is failure of bag integrity due to sharp instruments handled in or around the bag. Yet, this can be easily eliminated. Only materials should be used that can withstand forces applied during power morcellation, including instrument and tissue manipulation, as well as pressure and airflow caused by insufflation of the bag. In addition, bags should remain impermeable to tissue cells under these conditions. Furthermore, gynecologists should be aware of bag integrity failures when using their instruments near the bag [5, 15] Finally, the design of containment bags should be able to accommodate the camera, morcellator, and possibly a third instrument in the bag. The intentional puncture of retrieval bags has already been shown as possible cause for tissue leakage during in-bag morcellation [7]. By avoiding this puncture, 2 of the 10 (LM) and 2 of 6 (LH) main causes for spillage can be eliminated (Table 2). To our knowledge, of the currently available morcellation bags only one claims to meet the above-mentioned requirements. This bag was recently permitted to market by the FDA [16].

There are some considerations when interpreting the results from our study. Firstly, the consequence of micro-spillage is unclear as discussed. Nevertheless, there is increasing evidence that disease outcome is negatively influenced after (all types of) morcellation [17–20]. Next, there are limitations regarding the HFMEA.

This method was considered too time consuming in some studies and concerns were raised regarding the external validity of the prioritizing of hazards in the HFMEA [21, 22]. The severity and probability of a potential hazard may not always be known and must therefore be estimated by the team. In addition, hazards may be overlooked altogether. On the other hand, the identification of all (sub)steps and hazards of a procedure was reliable and reproducible [22]. In our opinion, this is the most important component of the HFMEA as the identification of such hazards, regardless of prioritization, allows adjustments and improvements of the assessed technology. Finally, although the HFMEA was time consuming, the time we spent is insignificant in relation to the time needed to repair adverse events that could have been prevented with a thorough prospective evaluation. Indeed, it can be questioned if a thorough risk analysis of power morcellation would have resulted in early warnings regarding the spill of malignant tissue being taken more seriously [23–27].

In conclusion, this study has demonstrated that the HFMEA is a valuable part of a prospective risk inventory of new surgical technology. Using in-bag morcellation as a template, the HFMEA has provided important new insights regarding potential weaknesses of this technique that were previously not recognized, even though in-bag morcellation is proposed as the new standard for morcellation. In addition, the recommendations that resulted from the HFMEA could be easily implemented in daily clinical practice.

## References

[1] Orde van Medisch Specialisten, Zorginstituut Nederland, Kennisinstituut van Medisch Specialisten (2017) Leidraad Nieuwe Interventies in de Klinische praktijk

[2] US Department of Defense (1949) Procedures for performing a failure mode, effects and criticality analysis. MIP-P-1629

[3] DeRosier J, Stalhandske E, Bagian JP, Nudell T (2002). Using health care failure mode and effect analysis: the VA National Center for Patient Safety’s prospective risk analysis system. Jt Comm J Qual Improv.

[4] Guedon AC, Wauben LS, van der Eijk AC, Vernooij AS, Meeuwsen FC, van der Elst M, Hoeijmans V, Dankelman J, van den Dobbelsteen JJ (2016). Where are my instruments? Hazards in delivery of surgical instruments. Surg Endosc.

[5] Cohen SL, Greenberg JA, Wang KC, Srouji SS, Gargiulo AR, Pozner CN, Hoover N, Einarsson JI (2014). Risk of leakage and tissue dissemination with various contained tissue extraction (CTE) techniques: an in vitro Pilot Study. J Minim Invasive Gynecol.

[6] Cohen SL, Einarsson JI, Wang KC, Brown D, Boruta D, Scheib SA, Fader AN, Shibley T (2014). Contained power morcellation within an insufflated isolation bag. Obstet Gynecol.

[7] Cohen SL, Morris SN, Brown DN, Greenberg JA, Walsh BW, Gargiulo AR, Isaacson KB, Wright K, Srouji SS, Anchan RM, Vogell AB, Einarsson JI (2015). Contained tissue extraction using power morcellation: prospective evaluation of leakage parameters. Am J Obstet Gynecol.

[8] Einarsson JI, Cohen SL, Fuchs N, Wang KC (2014). In-Bag Morcellation. J Minim Invasive Gynecol.

[9] Habraken MM, Van der Schaaf TW, Leistikow IP, Reijnders-Thijssen PM (2009). Prospective risk analysis of health care processes: a systematic evaluation of the use of HFMEA in Dutch health care. Ergonomics.

[10] Ben-Arie A, Tamir S, Dubnik S, Gemer O, Ben SA, Dgani R, Peer G, Barnett-Griness O, Lavie O (2008). Does hysteroscopy affect prognosis in apparent early-stage endometrial cancer?. Int J Gynecol Cancer.

[11] Devore GR, Schwartz PE, Morris JM (1982). Hysterography: a 5-year follow-up in patients with endometrial carcinoma. Obstet Gynecol.

[12] Yazbeck C, Dhainaut C, Batallan A, Benifla JL, Thoury A, Madelenat P (2005). Diagnostic hysteroscopy and risk of peritoneal dissemination of tumor cells. Gynecol Obstet Fertil.

[13] Toubia T, Moulder JK, Schiff LD, Clarke-Pearson D, O’Connor SM, Siedhoff MT (2016). Peritoneal washings after power morcellation in laparoscopic myomectomy: a Pilot Study. J Minim Invasive Gynecol.

[14] Sandberg EM, van den Haak L, Bosse T, Jansen FW (2016). Disseminated leiomyoma cells can be identified following conventional myomectomy. BJOG.

[15] Solima E, Scagnelli G, Austoni V, Natale A, Bertulessi C, Busacca M, Vignali M (2015). Vaginal uterine morcellation within a specimen containment system: a study of bag integrity. J Minim Invasive Gynecol.

[16] Food US, Administration D (2016) FDA allows marketing of first-of-kind tissue containment system for use with certain laparoscopic power morcellators in select patients

[17] Bogani G, Cliby WA, Aletti GD (2014). Impact of Morcellation on Survival Outcomes of Patients with Unexpected Uterine Leiomyosarcoma: a systematic review and meta-analysis. Gynecol Oncol.

[18] Park JY, Park SK, Kim DY, Kim JH, Kim YM, Kim YT, Nam JH (2011). The impact of tumor morcellation during surgery on the prognosis of patients with apparently early uterine leiomyosarcoma. Gynecol Oncol.

[19] George S, Barysauskas C, Serrano C, Oduyebo T, Rauh-Hain JA, del Carmen MG, Demetri GD, Muto MG (2014). Retrospective cohort study evaluating the impact of intraperitoneal morcellation on outcomes of localized uterine leiomyosarcoma. Cancer.

[20] Raspagliesi F, Maltese G, Bogani G, Fuca G, Lepori S, De IP, Perrone M, Scambia G, Cormio G, Bogliolo S, Bergamini A, Bifulco G, Casali PG, Lorusso D (2017). Morcellation worsens survival outcomes in patients with undiagnosed uterine leiomyosarcomas: a retrospective MITO group study. Gynecol Oncol.

[21] Kessels-Habraken M, Van der Schaaf T, De JJ, Rutte C, Kerkvliet K (2009). Integration of prospective and retrospective methods for risk analysis in hospitals. Int J Qual Health Care.

[22] Franklin BD, Shebl NA, Barber N (2012). Failure mode and effects analysis: too little for too much?. BMJ Qual Saf.

[23] Pelosi M (1997). Transvaginal uterine morcellation with unsuspected adenocarcinoma of the endometrium. Int J Gynaecol Obstet.

[24] Takamizawa S, Minakami H, Usui R, Noguchi S, Ohwada M, Suzuki M, Sato I (1999). Risk of complications and uterine malignancies in women undergoing hysterectomy for presumed benign leiomyomas. Gynecol Obstet Investig.

[25] Miller CE (2001). Methods of tissue extraction in advanced laparoscopy. Curr Opin Obstet Gynecol.

[26] Sinha RY, Joshi KM, Warty NR, Frey B (2000). Morcellation in the bag: the superior solution to avoid spillage. Gynaecol Endosc.

[27] Einstein MH, Barakat RR, Chi DS, Sonoda Y, Alektiar KM, Hensley ML, Abu-Rustum NR (2008). Management of uterine malignancy found incidentally after supracervical hysterectomy or uterine morcellation for presumed benign disease. Int J Gynecol Cancer.

